# Characterization of Uptake and Internalization of Exosomes by Bladder Cancer Cells

**DOI:** 10.1155/2014/619829

**Published:** 2014-01-19

**Authors:** Carrie A. Franzen, Patricia E. Simms, Adam F. Van Huis, Kimberly E. Foreman, Paul C. Kuo, Gopal N. Gupta

**Affiliations:** ^1^Department of Urology, Loyola University Chicago, Maywood, IL 60153, USA; ^2^FACS Core Facility, Research Services, Loyola University Chicago, Maywood, IL 60153, USA; ^3^Stritch School of Medicine, Loyola University Chicago, Maywood, IL 60153, USA; ^4^Oncology Institute, Loyola University Chicago, Maywood, IL 60153, USA; ^5^Department of Pathology, Loyola University Chicago, Maywood, IL 60153, USA; ^6^Department of Surgery, Loyola University Chicago, Maywood, IL 60153, USA

## Abstract

Bladder tumors represent a special therapeutic challenge as they have a high recurrence rate requiring repeated interventions and may progress to invasive or metastatic disease. Exosomes carry proteins implicated in bladder cancer progression and have been implicated in bladder cancer cell survival. Here, we characterized exosome uptake and internalization by human bladder cancer cells using Amnis ImageStreamX, an image cytometer. Exosomes were isolated by ultracentrifugation from bladder cancer culture conditioned supernatant, labeled with PKH-26, and analyzed on the ImageStreamX with an internal standard added to determine concentration. Exosomes were cocultured with bladder cancer cells and analyzed for internalization. Using the IDEAS software, we determined exosome uptake based on the number of PKH-26+ spots and overall PKH-26 fluorescence intensity. Using unlabeled beads of a known concentration and size, we were able to determine concentrations of exosomes isolated from bladder cancer cells. We measured exosome uptake by recipient bladder cancer cells, and we demonstrated that uptake is dose and time dependent. Finally, we found that uptake is active and specific, which can be partially blocked by heparin treatment. The characterization of cellular uptake and internalization by bladder cancer cells may shed light on the role of exosomes on bladder cancer recurrence and progression.

## 1. Introduction

Bladder cancer is the fourth most common noncutaneous malignancy in the US. Bladder cancer incidence has been steadily increasing, with minimal progress made in detection and risk stratification. Furthermore, the risk of recurrence and progression remains significant [1]. Thus, there is an urgent need to identify novel biomarkers and mechanisms of bladder cancer progression for therapeutic targeting [2].

Exosomes are microvesicles 30–100 nm in size, which are secreted from cells and contain proteins, mRNA, and miRNA. Studies have shown that bladder cancer cell lines shed exosomes containing proteins important for tumor progression [3–5], and these exosomes inhibit tumor cell apoptosis through Akt and ERK pathways [6]. Intravesical shedding of bladder tumor exosomes may promote the multifocality or progression of bladder lesions, thus implicating exosomes in the recurrence and progression of bladder cancer. Therefore, a better understanding and characterization of bladder cancer-shed exosome uptake by recipient bladder cancer cells and their downstream effects are needed.

Several tools are currently used to quantitate exosomes and visualize uptake, including Nanosight, flow cytometers, and confocal microscopes. However, there are limitations to analyzing exosomes with any of these methods. Nanoparticle tracking analysis and flow cytometry cannot measure uptake, whereas confocal microscopy [7, 8] is subjective, is time consuming, and allows for a limited number of cells to be analyzed.

To overcome these challenges, we employed the Amnis ImageStreamX, an image cytometer, as a novel method for both quantitating exosomes and measuring uptake by recipient bladder cancer cells, thus overcoming the limitations of the current tools. Image cytometry provides area of brightfield and fluorescence intensity measurements like a flow cytometer, and it can quantitate morphological features as seen through microscopy using image analysis software, IDEAS. We quantitated membrane dye labeled exosomes isolated from human bladder cancer cells and characterized uptake by recipient bladder cancer cells. We elucidated several aspects of exosome uptake, including internalization, in a statistically valid and reproducible manner.

## 2. Materials and Methods

### 2.1. Cell Culture

SW780 and UMUC3 human bladder cancer cell lines were purchased from ATCC and cultured in DMEM containing 10% fetal bovine serum, 100 units/mL penicillin, 100 *μ*g/mL streptomycin, and 2 mmol/L L-glutamine.

### 2.2. Reagents and Antibodies

PKH26 and heparin sodium salt were from Sigma Aldrich. CD63 and HSP70 polyclonal antibodies and horseradish peroxidase-conjugated goat anti-rabbit secondary antibody were from System Biosciences. Calnexin polyclonal and FITC-conjugated Coxsackie and Adenovirus Receptor (CAR) antibodies were from Santa Cruz Biotechnology. Alexa Fluor 488 Phalloidin was from Life Technologies.

### 2.3. Exosome Isolation

Exosomes were isolated from SW780 cell conditioned media by differential centrifugation, as previously described [9]. Conditioned media were collected after 48 hours from 8 × P150 plates of SW780 cells. The conditioned media were centrifuged at 300 ×g for 10 minutes to remove contaminating cells. The supernatant was collected and centrifuged at 2000 ×g for 10 minutes to pellet dead cells. The supernatant was then filtered through a 0.22 *μ*m filter and ultracentrifuged at 100,000 ×g for 70 minutes. The pellets were washed with PBS, pooled, and ultracentrifuged at 100,000 ×g for 70 minutes. The final pellet was resuspended in PBS or RIPA buffer.

### 2.4. Transmission Electron Microscopy (TEM)

Exosomes were imaged by TEM as previously described [10] and then imaged using a Hitachi H-600 TEM operating at 60 kV.

### 2.5. Immunoblotting

SW780-derived exosomes or SW780 cells were lysed in RIPA buffer containing protease inhibitors and proteins were resolved using SDS-PAGE. After transfer to a PVDF membrane, standard immunoblot analysis was performed.

### 2.6. Exosome Labeling

Exosomes were labeled with PKH26, according to the manufacturer's protocol, with some modifications. Briefly, exosome pellets were resuspended in 1 mL Diluent C. Separately, 1 mL Diluent C was mixed with 4 *μ*L PKH26. The exosome suspension was mixed with the stain solution and incubated for four minutes. The labeling reaction was stopped by adding an equal volume of 1% BSA. Labeled exosomes were ultracentrifuged at 100,000 ×g for 70 minutes, washed with PBS, and ultracentrifuged again.

### 2.7. Cell Surface Staining

SW780 or UMUC3 cells were cocultured with SW780-derived exosomes. Cells were detached by calcium sequestration under physiological conditions and surface stained with CAR-FITC according to standard protocols [11]. Labeled cells were analyzed on the ImageStreamX. Where indicated, cells or labeled exosomes were pretreated with 10 *μ*g/mL heparin for 30 minutes, followed by coincubation of exosomes with cells.

### 2.8. Amnis ImageStreamX

Acquisition was performed using ImageStreamX Imaging Flow Cytometer (Amnis Corporation, Seattle, WA) equipped with INSPIRE software. A 60x magnification was used for all samples. A minimum of 10,000 cells were analyzed for each sample. Data analysis was performed using the IDEAS software (Amnis Corporation). FITC and PKH26 were excited with a 100 mW of 488 nm argon laser. FITC and PKH26 fluorescence were collected on channel two (505–560 nm) and channel three (560–595 nm), respectively. Intensity adjusted brightfield images were collected on channel four.

### 2.9. Sensitivity of Amnis ImageStream

The lower limits of fluorescence detection were analyzed using Quantum FITC-5 MESF (Bangs Laboratories, Fishers, IN). Beads containing 2,264–860048 MESF (Molecules of Equivalent Soluble Fluorochrome) were analyzed on the ImageStream using instrument settings identical to assay conditions. Beads with 15033 MESF were distinct from background. The lower limits of detection for size and fluorescence were determined using Sphero Nano Fluorescence beads (Spherotech, Lake Forest, IL) with bead sizes 220–1340 nm. Brightfield area and total fluorescence intensity were calculated using IDEAS software.

### 2.10. IDEAS Analysis

Data analysis was performed using the IDEAS software (Amnis Corporation). Data were compensated using a compensation matrix generated using singly stained samples. The compensated data was then gated using the following pattern. First, a focus gate was determined to eliminate cells that were not in the field of focus; second, the focused cells were gated to eliminate doublets and debris. Gated data was used to generate histograms measuring fluorescence intensity (sum of all pixels in an image), median pixel intensity (pixel intensity value separating the brighter half from the less bright half), and max pixel intensity (intensity of the brightest pixels in an image) for each sample. The IDEAS software contains wizards to measure internalization and count spots. For internalization studies, the cell surface marker CAR and exosomal marker PKH26 were identified and the software generated masks for these signals, compared those signals, and generated an index of colocalization. For the spot counting wizard, subpopulations of cells with low and high exosomes numbers were manually identified as truth sets, and the software used these data sets to determine the number of exosome spots per cell. The data were reported as a histogram of spots for each sample, and the median spot number was reported. Percent uptake was calculated by multiplying the number of spots per cell by the total cell number. This value (total number of exosomes taken up by cells) was then divided by the amount of exosomes added to the cells (which was based on the concentration determination from the ImageStreamX).

### 2.11. Exosome Quantification

Exosome preparations were serially diluted, and an internal standard counting bead was added to each sample. Sample data was acquired on the ImageStreamX. The number of beads was determined using area and aspect ratio plots. The number of exosomes was determined using a PKH26 intensity plot. The ratio of beads to exosomes determined the concentration of exosomes/mL.

### 2.12. Deconvolution Microscopy

SW780 or UMUC3 cells were cocultured with exosomes derived from SW780 cells for four hours. Cells were fixed with 4% paraformaldehyde, permeabilized with 0.1% Triton X-100, and stained with phalloidin. Actin staining was used to create a mask to determine whether exosomes were inside or on the cell surface. Images were collected with a DeltaVision microscope (Applied Precision) equipped with a digital camera (CoolSNAP HQ; Photometrics), using a 1.4-numerical aperture 100× objective lens, and deconvolved with SoftWoRx deconvolution software (Applied Precision). Actin masks were applied to deconvolved images by creating an algorithm within the Create Surface Mode of the Imaris software package (Bitplane).

## 3. Results

### 3.1. Isolation and Characterization of Human Bladder Cancer Exosomes

**Figure 1:**
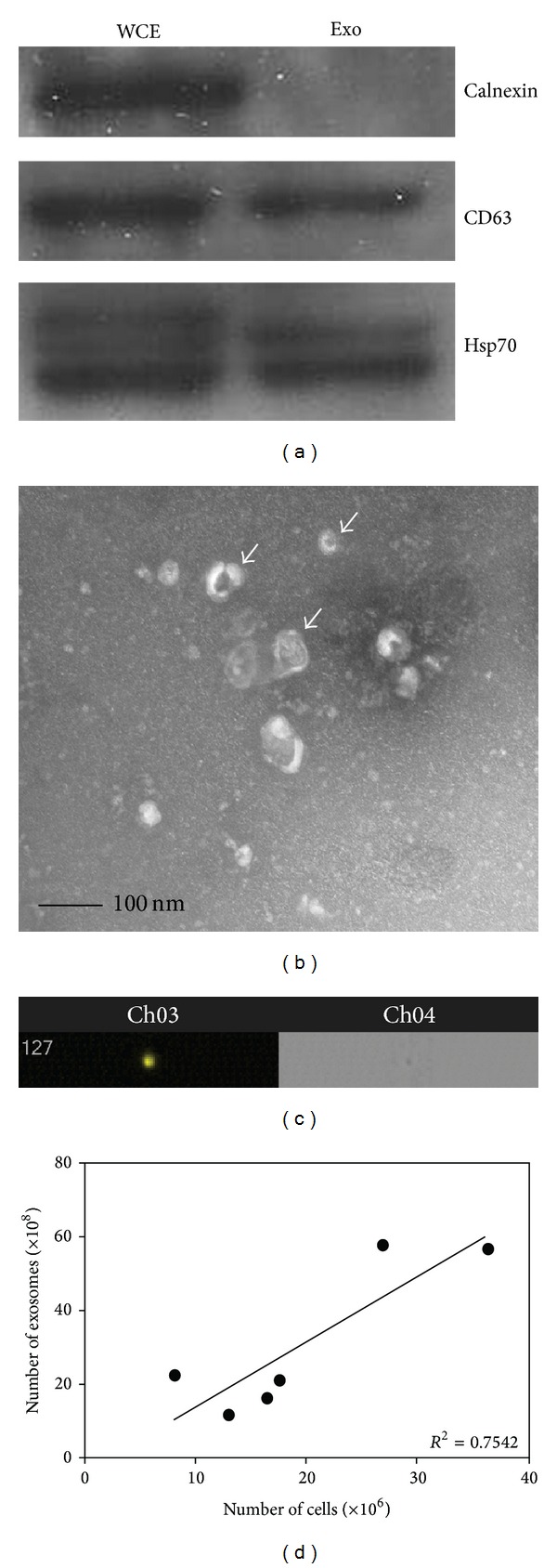
Isolation and characterization of exosomes from SW780 bladder cancer cells. (a) Representative Western blot of exosomal markers in exosomes (exo) isolated from SW780 whole cell extract (WCE) and exosomes. Western blots were repeated three times. (b) Morphological characterization of exosomes isolated from SW780 cells by transmission electron microscopy at 40,000x magnification. TEM was performed at least three times. A representative image is shown. Scale bar shows 100 nm. Arrows indicate exosomes. (c) Representative image of labeled exosomes from SW780 cell exosomes, which were not detectable using the brightfield signal, but were using the PKH26 signal. (d) Correlation between SW780 cell number and number of exosomes secreted by these cells. *R* ^2^ value was calculated using GraphPad Prism software. Data is represented as average ± SEM.

Exosomes are difficult to visualize and quantitate due to their small size. To address this, exosomes were isolated from SW780 cells and labeled with PKH26 and their identity was confirmed by Western blot analysis of specific markers. Calnexin, an endoplasmic reticulum marker, was only detectable in whole cell extracts, demonstrating that the exosome preparations were not contaminated with other vesicles or cellular components [12]. CD63, a tetraspanin, was present in both the whole cell extract and exosome preparation (Figure 1(a)). TEM showed that 50–100 nm vesicles were isolated, with typical exosome morphology (Figure 1(b)). PKH26-labeled exosomes were analyzed on the ImageStreamX. The limits of fluorescence intensity for the ImageStream were determined with a linear relationship between fluorescence intensity and concentration throughout the detectable range confirmed. PKH26 intensity is proportional to size of the particle. To determine whether the ImageStream was able to detect fluorescence from particles as small as exosomes, analysis of fluorescent nanoparticles was performed. Fluorescence intensity correlated well with size (see Supplementary Figure  1 in Supplementary Material available online at http://dx.doi.org/10.1155/2014/619829). Exosomes were detectable using the PKH26 signal but not the brightfield signal (Figure 1(c)). Exosomes were quantitated using an internal standard to allow for concentration determination. We found a strong correlation between cell number and number of exosomes isolated from the cells (Figure 1(d)).

### 3.2. Exosome Uptake in Bladder Cancer Cells Is Dose and Time Dependent

**Figure 2:**
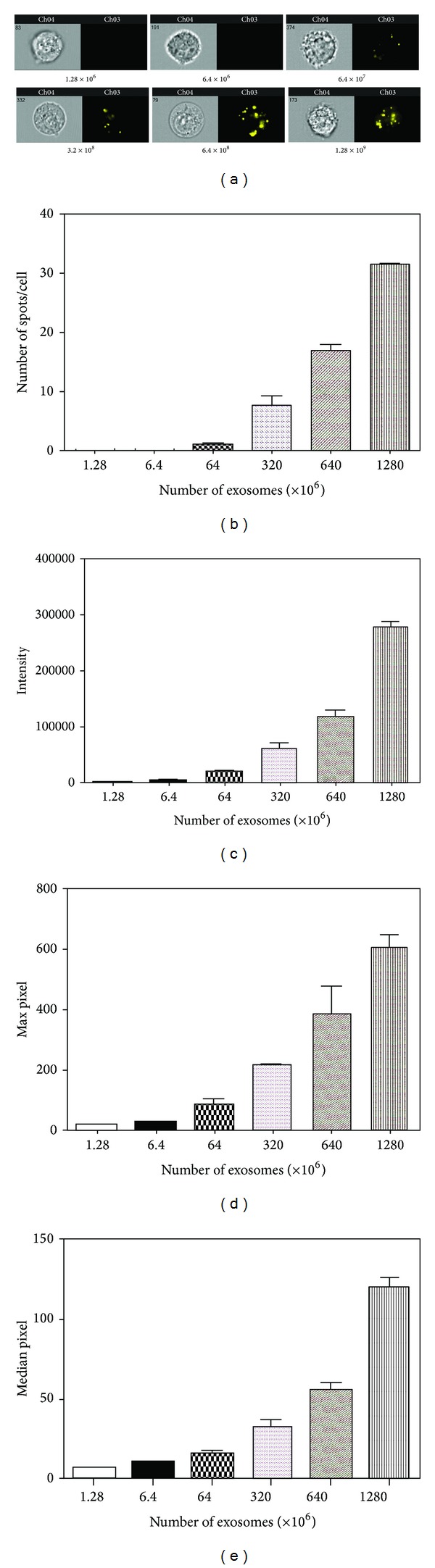
Exosome uptake in bladder cancer cells is dose dependent. Labeled exosomes isolated from SW780 cells were incubated at various concentrations with SW780 cells for 4 hours. This experiment was performed at least three times, with data from a representative experiment shown. A minimum of 10,000 cells were analyzed for each sample. (a) Representative images from the Amnis ImageStreamX. (b) Spot count data using the IDEAS software. (c) Fluorescence intensity of labeled exosomes, quantitated by the IDEAS software. (d) Max pixel quantitation of labeled exosomes. (e) Median pixel quantitation of labeled exosomes. Data is represented as average ± SEM.

In order to exert effects on cells, exosomes must be taken up by them. Thus, we investigated exosome uptake kinetics in bladder cancer cells. Increasing concentrations of PKH26-labeled SW780-derived exosomes were added to cultured SW780 cells and incubated for 4 hours. SW780 cells were labeled with the surface marker CAR and analyzed on the ImageStreamX (Figure 2(a)). Exosome uptake was dose dependent, as there was increased spot number (Figure 2(b)), total fluorescence, max pixel, and median pixel intensities of PKH26 (Figures 2(c)–2(e)) with increasing exosome concentrations.

**Figure 3:**
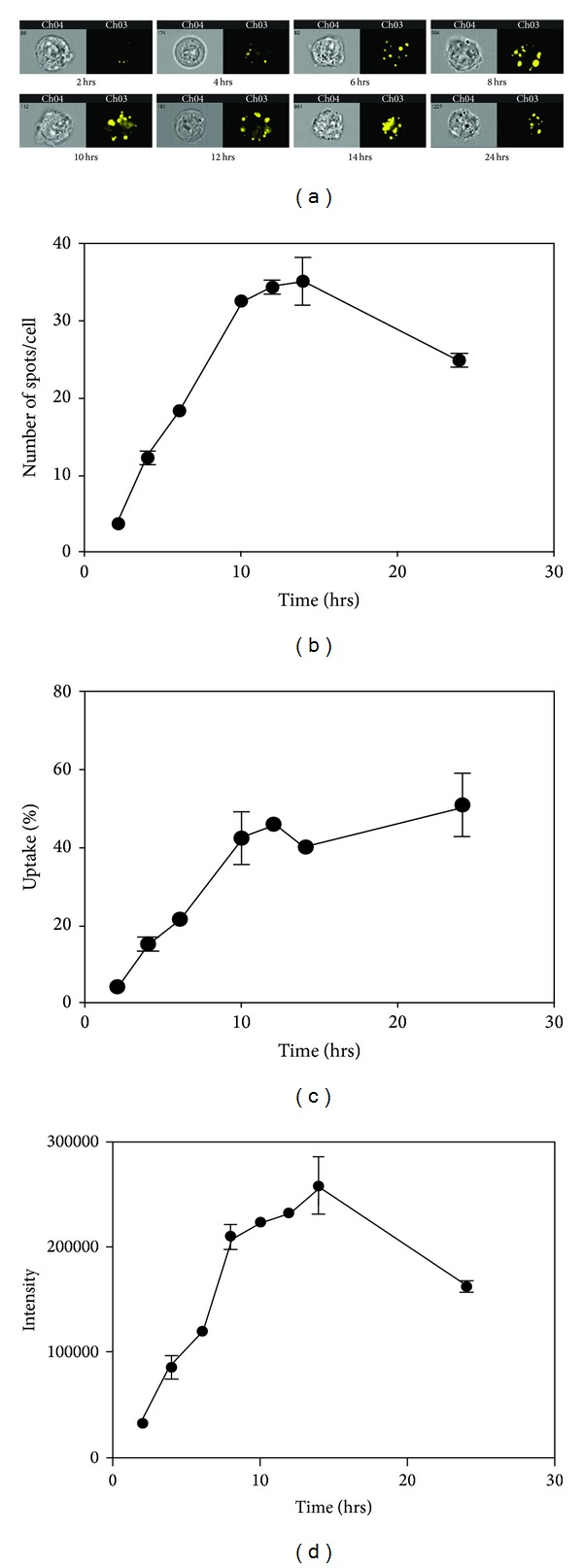
Exosome uptake in bladder cancer cells increases with longer incubation times. Labeled SW780 exosomes were incubated with SW780 cells for the indicated times. This experiment was performed at least three times, with data from a representative experiment shown. A minimum of 10,000 cells were analyzed for each sample. (a) Representative images. (b) Spot count of internalized labeled exosomes. (c) Calculation of percent uptake based on the number of exosomes added, the number of spots per cell, and the total number of cells. (d) Fluorescence intensity of the labeled exosomes. Data is represented as average ± SEM.

Furthermore, we observed increased uptake with longer incubation times (Figure 3(a)). Spot count analysis demonstrated increasing exosome numbers in cells as incubation time increased, with saturation around 14 hours (Figure 3(b)). When uptake efficiency was calculated (total number of exosomes taken up by cells divided by amount of exosomes added to cells), we found that, although spot count leveled off after 14 hours, exosomes continued to be taken up by cells as late as 24 hours (Figure 3(c)). We also demonstrated increased fluorescence intensity of PKH26-labeled exosomes with longer incubation times, with saturation around 14 hours (Figure 3(d)).

### 3.3. Exosomes Are Internalized by Bladder Cancer Cells

**Figure 4:**
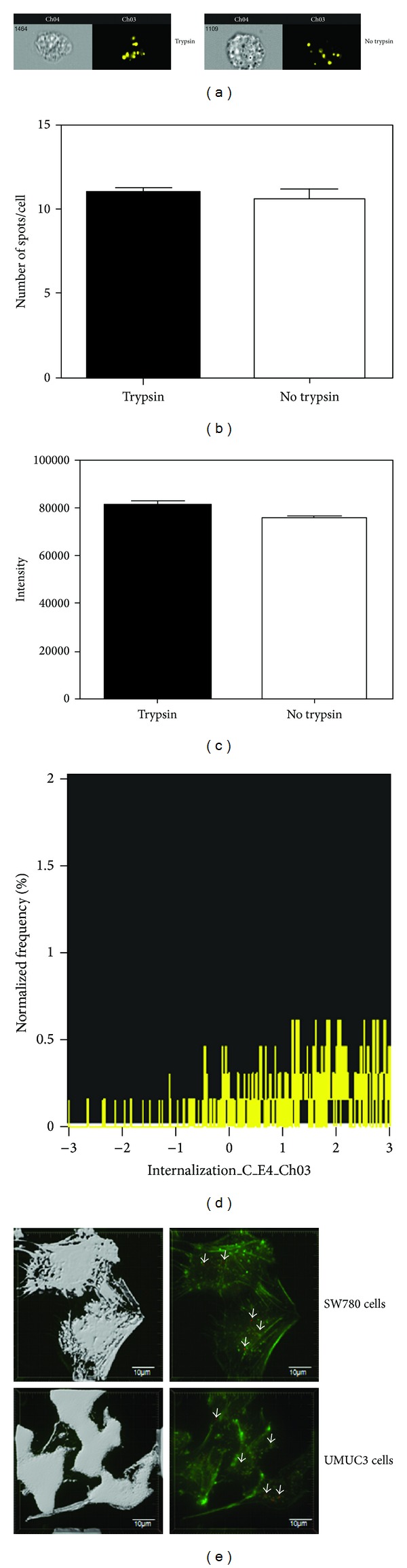
Exosomes are internalized by bladder cancer cells. SW780 cells were cultured with labeled exosomes for 4 hours, followed by detachment using either trypsin or calcium sequestration and labeling with CAR. This experiment was performed at least three times, with data from a representative experiment shown. A minimum of 10,000 cells were analyzed for each sample. (a) Representative images. (b) Spot count of labeled exosomes. (c) Fluorescence intensity of labeled exosomes. (d) Internalization wizard, measuring the ratio of the intensity inside the cell to the intensity of the entire cell. (e) Deconvolution microscopy with an actin (green) and PKH26-labeled exosomes (red). Actin staining was used as a cell marker in order to create a mask to determine whether exosomes were inside the cell or on the cell surface. The actin mask (left) shows that the exosomes are internalized. Arrows indicate exosomes. Data is represented as average ± SEM.

Internalization of exosomes is one mechanism of cargo delivery to recipient cells. We determined if exosomes were internalized or attached to the surface of recipient bladder cancer cells. Exosomes associated with cells were trypsin insensitive, as shown by spot count and fluorescence intensity, suggesting that they were not attached to the cell surface (Figures 4(a)-4(b)). The IDEAS software internalization wizard verified higher fluorescence intensity inside cells (PKH26) as compared to the fluorescence intensity of the entire cell (CAR-FITC surface staining), suggesting that exosomes were internalized (Figure 4(d)). Finally, we performed deconvolution microscopy using an actin mask. When the actin mask was on, exosomes were not visible; however, when the actin mask was removed, exosomes became visible, confirming that exosomes were internalized by cells (Figure 4(e)).

### 3.4. Overnight Storage of Exosomes at 4°C or −20°C Does Not Affect Uptake

**Figure 5:**
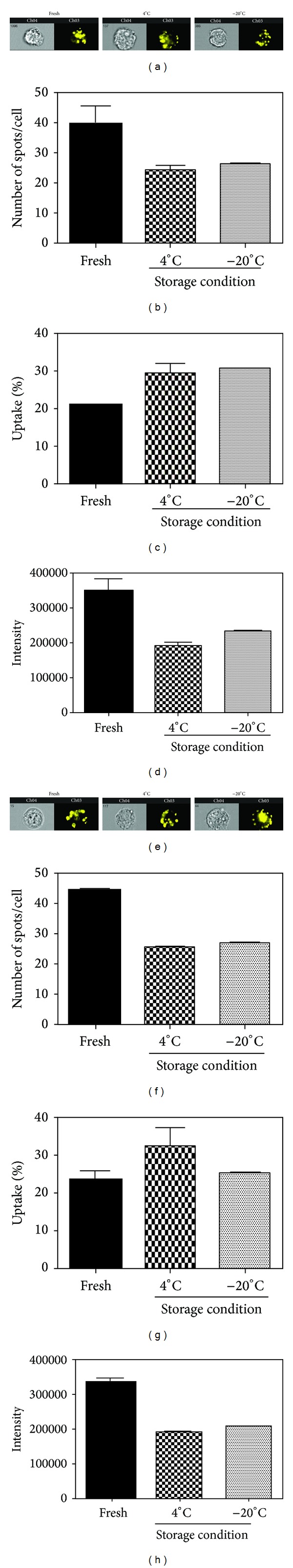
Overnight storage of exosomes at 4°C or −20°C does not affect uptake. SW780 cells were incubated for 4 hours with freshly isolated exosomes or exosomes that were stored overnight at 4°C or −20°C. This experiment was performed at least three times, with data from a representative experiment shown. A minimum of 10,000 cells were analyzed for each sample. (a) and (e) Representative images of SW780 cells (a) and UMUC3 cells (e). (b) and (f) Spot count for SW780 cells (b) and UMUC3 cells (f). (c) and (g) Fluorescence intensity of labeled exosomes taken up by SW780 (c) or UMUC3 (g) cells. (d) and (h) Percent uptake calculation for SW780 (d) or UMUC3 (h) cells. Data is represented as average ± SEM.

Nanoparticle tracking has demonstrated that exosome storage at 4°C caused aggregation, while short-term storage at −20°C had no effect [13]. We wanted to determine if overnight storage of exosomes impacted uptake. PKH26-labeled exosomes were either immediately added to SW780 and UMUC3 cells or stored overnight at 4°C or −20°C in PBS prior to adding to cells. Although we observed a slight decrease in spot count with overnight storage as compared to exosomes that were not stored (Figures 5(a)-5(b)), this difference could be explained by increased cell number due to an extra 24 hours of growth. This translates to decreased spot number per cell while still maintaining the same percent uptake of exosomes. There was no adverse effect on percent uptake by storing exosomes overnight (Figure 5(c)). The only consequence to overnight storage was decreased PKH26 fluorescence intensity (Figure 5(d)). When SW780 exosomes were added to another bladder cancer cell line, UMUC3, we observed the same trend (Figures 5(e)–5(h)).

### 3.5. Exosome Uptake Can Be Blocked at 4°C or with Heparin Treatment

**Figure 6:**
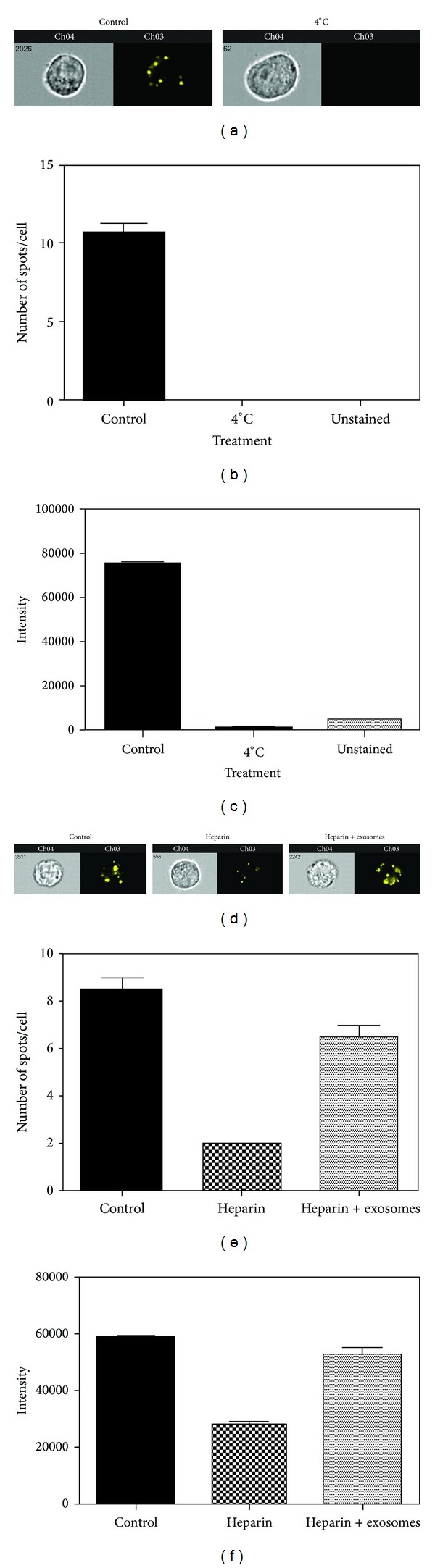
Exosome uptake can be blocked at 4°C or with heparin treatment. SW780 cells were cultured with labeled exosomes at 4°C for 4 hours (a–c) or pretreated with 10 *μ*g/mL heparin for 30 minutes, followed by incubation with exosomes for 4 hours (d–f). This experiment was performed at least three times, with data from a representative experiment shown. A minimum of 10,000 cells were analyzed for each sample. (a) and (d) Representative images. (b) and (e) Spot count of labeled exosomes. (c) and (f) Fluorescence intensity of labeled exosomes. Data is represented as average ± SEM.

Exosomes interact with and are taken up by cells in many ways, including receptor-mediated endocytosis, phagocytosis, micropinocytosis, or direct fusion with the plasma membrane [14–18]. To elucidate the mechanism of uptake in SW780 bladder cancer cells, we incubated exosomes with cells at either 4°C or 37°C. At 4°C, there were complete abrogation of uptake (Figures 6(a)-6(b)), as determined by spot count, and a substantial decrease in PKH26 fluorescence intensity (Figure 6(c)). This demonstrated that exosome uptake is a specific process and suggested that it is receptor-mediated. We next determined if exosome uptake could be inhibited with heparin, a competitive inhibitor of cell surface receptors dependent on Heparan Sulfate Proteoglycan (HSPG) coreceptors [19–21], including Syndecan, an HSPG shown to be important for exosome biogenesis [22]. We pretreated SW780 cells with 10 *μ*g/mL heparin, then cocultured with exosomes (Figure 6(d)). Pretreatment of SW780 cells with heparin partially decreased spot count (Figure 6(e)) and PKH26 fluorescence intensity due to decreased cellular uptake (Figure 6(f)). When exosomes, rather than cells, were pretreated with heparin, we observed a smaller decrease in spots/cell, suggesting that heparin may function primarily through inhibiting receptors on recipient cells rather than the exosomes themselves (Figures 6(d)–6(f)).

## 4. Discussion

Bladder tumors represent a unique therapeutic challenge as they have a high rate of local recurrence and multifocality requiring repeated interventions. They often progress to invasive or metastatic disease, which accounts for significant morbidity and mortality and requires aggressive treatment [23]. Exosomes may play a role in both recurrence and progression of bladder cancer through uptake by neighboring cells or other preexisting bladder cancer foci. This study characterizing release, uptake, and internalization of exosomes by human bladder cancer cell lines increases our understanding of the role exosomes play in bladder cancer pathogenesis with the ultimate goal of establishing exosomes as potential biomarkers and therapeutic targets.

To our knowledge, this is the first study using image cytometry to quantify exosomes and characterize cellular uptake and internalization in bladder cancer cells. Using the ImageStreamX, we established the minimal number of exosomes and incubation time required to achieve cellular uptake. We found a strong direct correlation between cell number and number of exosomes secreted by those cells providing a reproducible way to estimate the number of exosomes isolated from bladder cancer cells. This information is critical for future functional studies of known regulators of exosome secretion, such as Rab family [24], hypoxia [25], or changing calcium levels [26]. The ability to estimate the amount of exosomes isolated from bladder cancer cells, along with exosome protein quantitation, will be invaluable for analyzing phenotypic changes in functional assays.

We established that exosome uptake by recipient cells is both dose and time dependent. We further confirmed that exosomes were internalized. These findings demonstrate that exosomes and their cargo can be transferred between cells. This supports our initial hypothesis that intravesical shedding of exosomes from bladder tumors may promote the multifocality of bladder cancer or lead to progression of other bladder lesions. During our time course, we found that, at 24-hour incubation, there were fewer exosomes per cell than at 12 or 14 hours. However, when we calculated percent uptake, we discovered that exosomes were still being taken up by the cells. This may be due to cell division, as there were more cells at 24 hours. It is also possible that we measured less spots per cell because exosomes that had been taken up early by cells released their cargo into the cytoplasm and their membrane has either been incorporated into the plasma membrane or degraded.

We demonstrated that overnight storage at 4°C or −20°C did not impact exosome uptake. It has been shown that storing exosomes at 4°C for 24 hours or longer led to their aggregation [13]. However, storage at −20°C did not appear to have adverse effects on exosomes [13]. It would be interesting to determine if exosome uptake is affected by storage at 4°C or −20°C for 24 hours or more. As exosome isolation is laborious, the ability to store freshly isolated exosomes for later experimental use without detriment to uptake is a significant finding.

Our data suggest that, in SW780 bladder cancer cells, uptake occurs primarily through receptor-mediated endocytosis, in part through HSPGs. We found that HSPGs on recipient cells are primarily involved in the uptake process with those present on exosomes playing a minor role. Heparin treatment only partially blocked uptake suggesting that other receptors may be involved. Also, our deconvolution microscopy data shows that, while exosomes may bind to cells through cell-surface receptors, they are subsequently internalized. This is in agreement with other studies, which demonstrate that exosome uptake occurs through receptor-mediated endocytosis [27, 28]. It would be interesting to determine if blocking receptor-mediated endocytosis would inhibit exosome uptake in SW780 cells and if blocking both caveolin and HSPGs would synergistically abrogate cellular uptake of exosomes.

Image cytometry provides a streamlined way to both quantify isolated exosomes and visualize their uptake, shedding light on key properties of exosome biology. Use of the ImageStream allowed us to characterize exosome uptake by bladder cancer cells in a statistically valid and reproducible manner and will provide a platform for assaying effects of factors or chemicals on exosome uptake in future research. The ImageStream can be used to quantify exosomes in urine specimens from patients with bladder cancer and measure differential uptake of bladder cancer exosomes by normal bladder urothelial cells and bladder cancer cells.

Taken together, our novel method of characterization of cellular uptake and internalization by bladder cancer cells could become invaluable for understanding the role of exosomes on bladder cancer recurrence and progression. Furthermore, the ability to study the biology of exosome uptake in cancer cells in an unbiased, quantitative, and reproducible manner will enable standardization of exosome functional studies.

## Supplementary Material

Supplemental Figure 1. Sensitivity of Amnis ImageStream:
A. The lower limits of fluorescence detection were analyzed using Quantum FITC-5 **MESF** (Bangs Laboratories, Fishers, IN). Beads containing 2,264-860048 **MESF** (Molecules of Equivalent Soluble Fluorochrome) were analyzed on the ImageStream using instrument settings identical to assay conditions. Beads with 15033 **MESF** were distinct from background. B. The lower limits of detection for size and fluorescence were determined using Sphero Nano Fluorescence beads (Spherotech, Lake Forest, IL) with bead sizes 220-1340nm. Brightfield area and total fluorescence intensity were calculated using IDEAS software.
