# Supplementary material for: Characterization of Uptake and Internalization of Exosomes by Bladder Cancer Cells
Source: Biomed Res Int. 2014 Jan 19;2014:619829. doi: 10.1155/2014/619829 (PMC3915764; doi:10.1155/2014/619829)
Supplement: Supplementary file 1 — Supplemental Figure 1. Sensitivity of Amnis ImageStream: A. The lower limits of fluorescence detection were analyzed using Quantum FITC-5 MESF (Bangs Laboratories, Fishers, IN). Beads containing 2,264-860048 MESF (Molecules of Equivalent Soluble Fluorochrome) were analyzed on the ImageStream using instrument settings identical to assay conditions. Beads with 15033 MESF were distinct from background. B. The lower limits of detection for size and fluorescence were determined using Sphero Nano Fluorescence beads (Spherotech, Lake Forest, IL) with bead sizes 220-1340nm. Brightfield area and total fluorescence intensity were calculated using IDEAS software. [file 619829.f1.pdf]

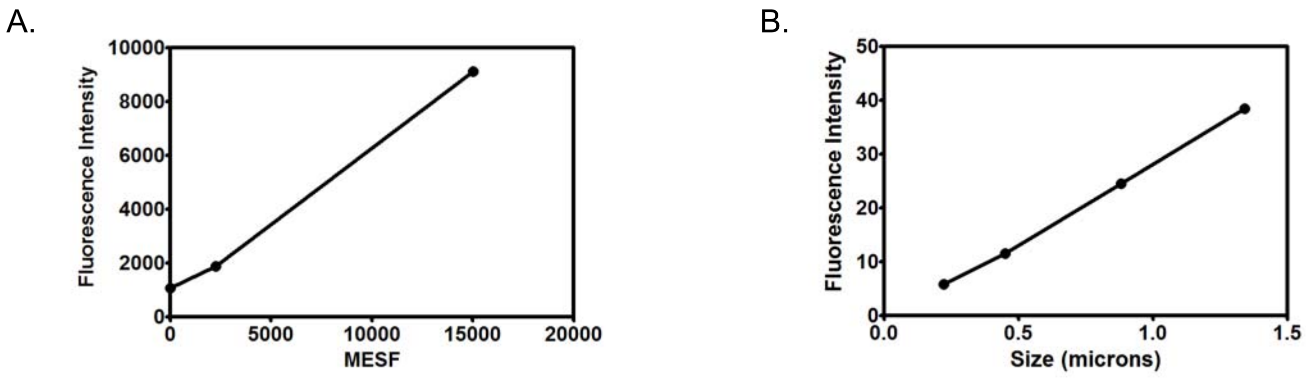

Supplemental Figure 1. Sensitivity of Amnis ImageStream. A. The lower limits of fluorescence detection were analyzed using Quantum FITC-5 MESF (Bangs Laboratories, Fishers, IN). Beads containing 2,264-860048 MESF (Molecules of Equivalent Soluble Fluorochrome) were analyzed on the ImageStream using instrument settings identical to assay conditions. Beads with 15033 MESF were distinct from background. B. The lower limits of detection for size and fluorescence were determined using Sphero Nano Fluorescence beads (Spherotech, Lake Forest, IL) with bead sizes 220-1340nm. Brightfield area and total fluorescence intensity were calculated using IDEAS software.
